# circ_0025033 promotes ovarian cancer development via regulating the hsa_miR-370-3p/SLC1A5 axis

**DOI:** 10.1186/s11658-022-00364-2

**Published:** 2022-10-22

**Authors:** Huiping Ma, Shuyun Qu, Yao Zhai, Xiaofeng Yang

**Affiliations:** 1 Department of Gynecology, First Affiliated Hospital of Xi’an Jiaotong University, No. 277 Yanta West Road, Xi’an, 710000 Shaanxi China; 2 Department of Gynecology, Gynaecology Hospital of Shaanxi Nuclear Industry, Xi’an, Shaanxi China

**Keywords:** Ovarian cancer, circ_0025033, hsa_miR-370-3p, *SLC1A5*

## Abstract

**Background:**

Circular RNAs (circRNAs) appear to be important modulators in ovarian cancer. We aimed to explore the role and mechanism of circ_0025033 in ovarian cancer.

**Methods:**

qRT-PCR was conducted to determine circ_0025033, hsa_miR-370-3p, and *SLC1A5* mRNA expression. Functional experiments were conducted, including Cell Counting Kit-8 (CCK-8), 5-ethynyl-2′-deoxyuridine (EdU), flow cytometry, transwell, tube formation, xenograft tumor model assay, western blot analysis of protein levels, and analysis of glutamine metabolism using commercial kits. Their predicted interaction was confirmed using dual-luciferase reporter and RNA pull-down.

**Results:**

circ_0025033 was upregulated in ovarian cancer; its knockdown induced proliferation, invasion, angiogenesis, glutamine metabolism, and apoptosis in vitro, and blocked tumor growth in vivo. circ_0025033 regulated ovarian cancer cellular behaviors via sponging hsa_miR-370-3p. In parallel, *SLC1A5* might abolish the anti-ovarian cancer role of hsa_miR-370-3p. Furthermore, circ_0025033 affected *SLC1A5* via regulating hsa_miR-370-3p.

**Conclusion:**

circ_0025033 might promote ovarian cancer progression via hsa_miR-370-3p/*SLC1A5*, providing an interesting insight into ovarian cancer tumorigenesis.

**Supplementary Information:**

The online version contains supplementary material available at 10.1186/s11658-022-00364-2.

## Highlights

- circ_0025033 knockdown inhibited ovarian cancer malignant behaviors.
- circ_0025033 served as a hsa_miR-370-3p sponge.
- hsa_miR-370-3p targeted *SLC1A5*.

**Supplementary Information:**

The online version contains supplementary material available at 10.1186/s11658-022-00364-2.

## Introduction

Ovarian cancer, a common gynecological malignancy, is considered to be a global health issue correlated with increased morbidity and mortality [1]. Many patients with ovarian cancer are not diagnosed until they reach an advanced stage because early lesions are not easy to detect [2]. Although tremendous efforts have been made in ovarian cancer treatment, the 5-year overall survival rate of patients with ovarian cancer is between 35% and 40% [3]. Hence, elucidating the molecular mechanism involved in ovarian cancer is crucial for discovering effective therapeutic targets.

Unlike linear RNAs, circular RNAs (circRNAs) have special covalently closed loop structures [4]. Widely expressed in the cytoplasm of eukaryotic cells, they often exert a role in specific patterns of tissue and developmental stages [5]. circRNAs are becoming attractive biomarkers of human diseases owing to their abundance and stability [6, 7]. Emerging evidence has revealed that dysregulated circRNAs are implicated in cancer initiation and development in a wide range of tumors [8–10]. Apart from that, some circRNAs participate in ovarian cancer processes by acting as tumor suppressors or promoters [11–13]. circ_0025033 is produced by the back-splicing of its parental forkhead box M1 (*FOXM1*) gene (located at chr12: 2966846–2983691), whose spliced mature sequence length is 3410 bp. *FOXM1* is an essential transcription regulator that might modulate multiple aspects of tumor progression [14, 15]. It has been confirmed that the downregulation of *FOXM1* could effectively hinder the proliferation, migration, and invasion of ovarian cancer cells in vitro [16, 17]. A previous report indicated that circ_0025033 upregulation might boost ovarian cancer development [18]. Yet, its function and mechanism remain largely unknown in ovarian cancer.

Research in the past decades has shown that circRNAs exert their functions via competitive endogenous RNAs (ceRNAs) through binding with miRNA response elements (MREs), thereby de-repressing target mRNA expression [19, 20]. As another type of ncRNA, miRNAs might achieve the regulation of target gene via binding to their 3′ untranslated region (UTR) [21]. miRNAs as anti-oncogenes or oncogenes regulate cellular biological activities in cancer progression [22–24]. A previous report showed that has_hsa_miR-370-3p could inhibit metastatic ability in ovarian cancer cells [25]. Moreover, a recent study indicated that SLC1A5 (also called ASCT2) plays a promoter role in ovarian cancer [26]. Here, by applying bioinformatics tools, we revealed that hsa_miR-370-3p possesses binding sites with circ_0025033 and *SLC1A5*. Hence, we further explored whether the regulatory impact of circ_0025033 ovarian cancer development is mediated via hsa_miR-370-3p–*SLC1A5*.

## Materials and methods

### Specimen collection

After obtaining informed consent, ovarian cancer tissue samples (*n* = 29) along with matched adjacent normal samples were harvested from sufferers of ovarian cancer at First Affiliated Hospital of Xi'an Jiaotong University. This research had acquired approval from the ethics committee of First Affiliated Hospital of Xi'an Jiaotong University.

### Cell culture and transfection

Stored under standard conditions (37 ℃; 5% CO_2_) in RPMI-1640 medium, human ovarian surface epithelial cells (HOSEPiC) cells (cat. no. #7310) were purchased from ScienCell Research Laboratories (Carlsbad, CA, USA). Two ovarian cancer cell lines (HEY; cat. no. CL-0671, OVCAR3; cat. no. CL-0178) were supplied by Procell (Wuhan, China), and two other cell lines (SKOV3; cat. no. BNCC338639, A2780; cat. no. BNCC351906) were obtained from BeNa Culture Collection (Beijing, China). Human umbilical vein endothelial cells (HUVECs; Procell) were grown in HUVEC-specific complete medium (Procell).

RiboBio (Guangzhou, China) provided circ_0025033 small interfering RNA (si-circ_0025033), circ_0025033-overexpressing RNA (circ_0025033), hsa_miR-370-3p mimic/inhibitor (hsa_miR-370-3p/anti-hsa_miR-370-3p), siRNA against *SLC1A5* (si-*SLC1A5*), *SLC1A5*-overexpressing RNA (*SLC1A5*), and controls (si-NC, pCD5-ciR, miR-NC, anti-miR-NC, si-con, and pcDNA), followed by Lipofectamine 3000 reagent treatment.

### Immunohistochemistry (IHC) analysis

After being fixed and embedded, tumor samples were cut into slices of 5 μm thickness. Then, Ki67 (ab15580; 1:200), SLC1A5 (ab237704; 1:500), c-Myc (ab32072; 1:200), or MMP9 (ab283575; 1:1000) at 4 ℃ were reacted with these sections overnight, which were further incubated with secondary antibody (ab205718; 1:2000). Finally, immunostaining images were obtained by microscope (Leica, Wetzlar, Germany). All antibodies were provided by Abcam (Cambridge, UK).

### qRT-PCR

Using TRIzol reagent (Invitrogen), the generated total RNA was reverse transcribed according to PrimeScript RT Reagent Kit. An miRNA reverse-transcription PCR kit was used to reverse transcribe has_mR-370-3p. Subsequently, cDNA amplification was implemented according to SYBR Green Master Mix (Roche, Shanghai, China) on CFX96 PCR equipment. After GAPDH or U6 normalization, the gene levels were evaluated via the 2^−ΔΔCt^ method. The primer sequences are listed in Table 1.

**Table 1:** The sequences of primers for RT-qPCR used in this study

Name	Sequence (5′–3′)
circ_0025033: forward	GGTGTGAGCCAGCTTGAGA
circ_0025033: reverse	GACGGGGGCTAGTTTTCATT
*FOXM1*: forward	TCTGCCAATGGCAAGGTCTCCT
*FOXM1*: reverse	CTGGATTCGGTCGTTTCTGCTG
hsa_miR-370-3p: forward	GTATGAGCCTGCTGGGGTGG
hsa_miR-370-3p: reverse	CAGTGCGTGTCGTGGAGT
*SLC1A5*: forward	TCCTCTTCACCCGCAAAAACCC
*SLC1A5*: reverse	CCACGCCATTATTCTCCTCCAC
U6: forward	CTCGCTTCGGCAGCACATATACT
U6: reverse	ACGCTTCACGAATTTGCGTGTC
GAPDH: forward	CTGACTTCAACAGCGACACC
GAPDH: reverse	TGCTGTAGCCAAATTCGTTGT

In addition, to validate the circular structure of this circRNA, the RNAs generated at 37 ℃ were reacted with RNase R (Seebio, Shanghai, China). Finally, RNA expression levels were assessed with qRT-PCR. Meanwhile, to check the distribution of circ_0025033 in ovarian cancer cells, the RNA from the nuclear and cytoplasmic fractions was distinguished using PARIS Kit (Invitrogen), followed by qRT-PCR analysis.

### Cell proliferation assays

After 48 h of transfection, we seeded SKOV3 and A2780 cells (5 × 10^3^ cells per well) into 96-well plates. After incubation for 24 h, Cell Counting Kit-8 (CCK-8) solution (10 μL; Beyotime, Jiangsu, China) was added to each well, followed by analysis via microplate reader.

After 48 h of transfection, 5-ethynyl-2′-deoxyuridine (EdU) assay was conducted, where tumor cells were cultured at 2 × 10^4^ cells per well. At 24 h post-incubation, EdU solution and paraformaldehyde (4%) were mixed with the cells into each well, which were next incubated with DAPI and analyzed using a microscope.

### Flow cytometry analysis

Annexin V-FITC and PI apoptosis detection kit purchased from Yeasen (Shanghai, China) detected apoptotic cells. After 48 h of transfection, we seeded SKOV3 and A2780 cells (2 × 10^5^ cells per well) into a six-well plate. After labeling with annexin V-FITC and PI, the solution was placed in a flow cytometer for analysis.

### Transwell assay

After 48 h of transfection, SKOV3 and A2780 cell suspension was introduced into the top chamber (24-well; Costar, Corning, NY, USA) precoated with Matrigel, while the bottom counterpart contained complete medium. Cells remaining bottom were fixed and stained after 24 h, and invasion pictures were obtained using a microscope (×100; Leica).

### Tube formation assay

Angiogenesis capability was assessed by tube formation assay. In brief, when transfected cells (SKOV3 and A2780) reached 80% confluence, the supernatant was collected as the conditioned medium. Twenty-four-well dishes were coated with Matrigel in each well at 37 ℃ to polymerize. Next, HUVECs were seeded into Matrigel-coated wells under different conditioned media for 6 h. Finally, results were analyzed under a microscope and using ImageJ.

### Western blot assay

Total protein was extracted using RIPA lysis buffer (Solarbio, Beijing, China). After quantification of total protein using BCA protein assay kit (Solarbio), protein samples were loaded onto SDS–PAGE prior to being immunoblotted onto PVDF membranes (Millipore, Billerica, MA, USA). After incubation with primary antibodies, these membranes were incubated for 2 h with a corresponding secondary antibody (ab205718; 1:5000; Abcam). The combined signals were analyzed using enhanced chemiluminescence (ECL) (Vazyme, Nanjing, China). The primary antibodies were purchased from Abcam: *SLC1A5* (ab237704; 1:1000), c-Myc (ab32072; 1:200), MMP9 (ab76003; 1:1000), and β-actin (1:2500; ab8227).

### Measurement of glutamine metabolism

According to the manufacturer’s protocols, glutamine consumption, α-ketoglutarate production, and glutamate production were determined according to glutamine assay, α-ketoglutarate assay, and glutamate assay kits (Abcam), respectively.

### Dual-luciferase reporter assay

These fragments of circ_0025033 or 3′ UTR of *SLC1A5* with or without putative binding sites of hsa_miR-370-3p were introduced via pmirGLO vector (YouBia, Changsha, China), generating WT/MUT-circ_0025033 and WT/MUT-*SLC1A5* 3′ UTR. Then, SKOV3 and A2780 cells were transfected with hsa_miR-370-3p/miR-NC and reporter vectors, followed by analysis using dual-luciferase reporter gene assay kit.

### RNA pull-down assay

After being transfected with biotinylated (bio)-hsa_miR-370-3p or miR-NC (GenePharma, Shanghai, China), harvested cells were lysed, followed by reaction with M-280 streptavidin (Invitrogen). Subsequently, beads were mixed with the biotinylated hsa_miR-370-3p for 10 min and analyzed via qRT-PCR.

### Tumor formation assay in vivo

Twelve 5-week-old BALB/c nude mice (female; Vital River, Beijing, China) were separated into two groups, followed by subcutaneous inoculation with A2780 cells with sh-circ_0025033 or sh-NC (RiboBio). Tumor volume was measured. After inoculation for 23 days, the excised tumors from these sacrificed mice were weighed and studied. Permission to perform this experiment was provided by the Animal Care and Use Committee of First Affiliated Hospital of Xi'an Jiaotong University.

### Statistical analysis

GraphPad Prism 7.0 software was used to process all data in this work, presented as mean ± standard deviation. *P*-value below 0.05 was considered statistically significant. Student’s *t*-test or one-way analysis of variance (ANOVA) was adopted for comparisons. Survival curve was analyzed by Kaplan–Meier method. Pearson’s correlation coefficient was used to determine correlations in expression.

## Results

### circ_0025033 was enhanced in ovarian cancer

**Fig. 1:**
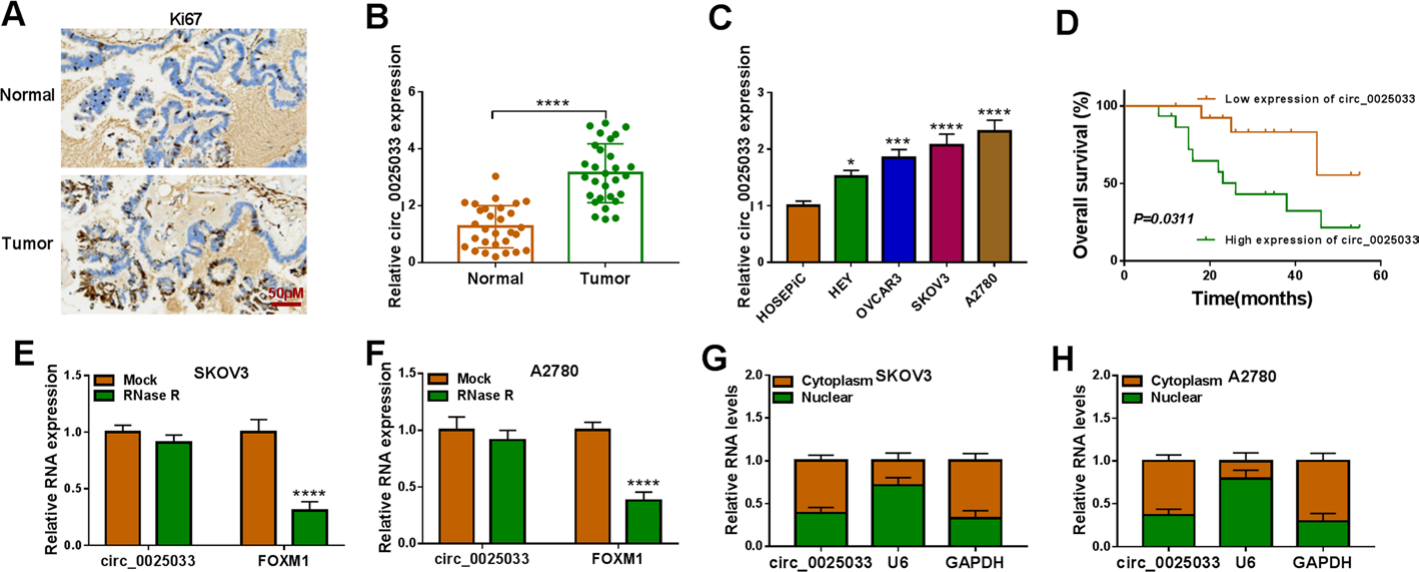
Overexpression of circ_0025033 in ovarian cancer. **A** IHC analysis detecting Ki67 content in tumor tissue. **B** and **C** qRT-PCR analysis of circ_0025033 content in tissue samples, HOSEPiC cells, and ovarian cancer cells (HEY, OVCAR3, SKOV3, and A2780). **D** Kaplan–Meier curves exhibiting survival rate of sufferers of ovarian cancer with high or low level of circ_0025033. **E** and **F** Expression of circ_0025033 and *FOXM1* determined via qRT-PCR after treatment of RNase R. **G** and **H** qRT-PCR analysis of circ_0025033 localization in tumor cells. *P < 0.05, ***P < 0.001, ****P < 0.0001

IHC analysis revealed higher Ki67 content in tumor tissue (Fig. 1A). circ_0025033 content was increased in ovarian cancer tissue and cells (HEY, OVCAR3, SKOV3, and A2780) (Fig. 1B and C). Among these ovarian cancer cells, circ_0025033 content was higher in SKOV3 and A2780 cells, so these two cell lines were selected for further analysis. Moreover, high level of circ_0025033 was predictive of poor prognosis in sufferers of ovarian cancer (Fig. 1D). In addition, linear *FOXM1* mRNA was degraded by RNase R, but there was no change in circ_0025033 level (Fig. 1E and F). Localization of circ_0025033 in tumor cells was determined. Figure 1G and H shows that circ_0011298 was prominently located in tumor cell cytoplasm. Taken together, the findings show that circ_0025033 was upregulated in ovarian cancer.

### circ_0025033 absence inhibits ovarian cancer cell development

**Fig. 2:**
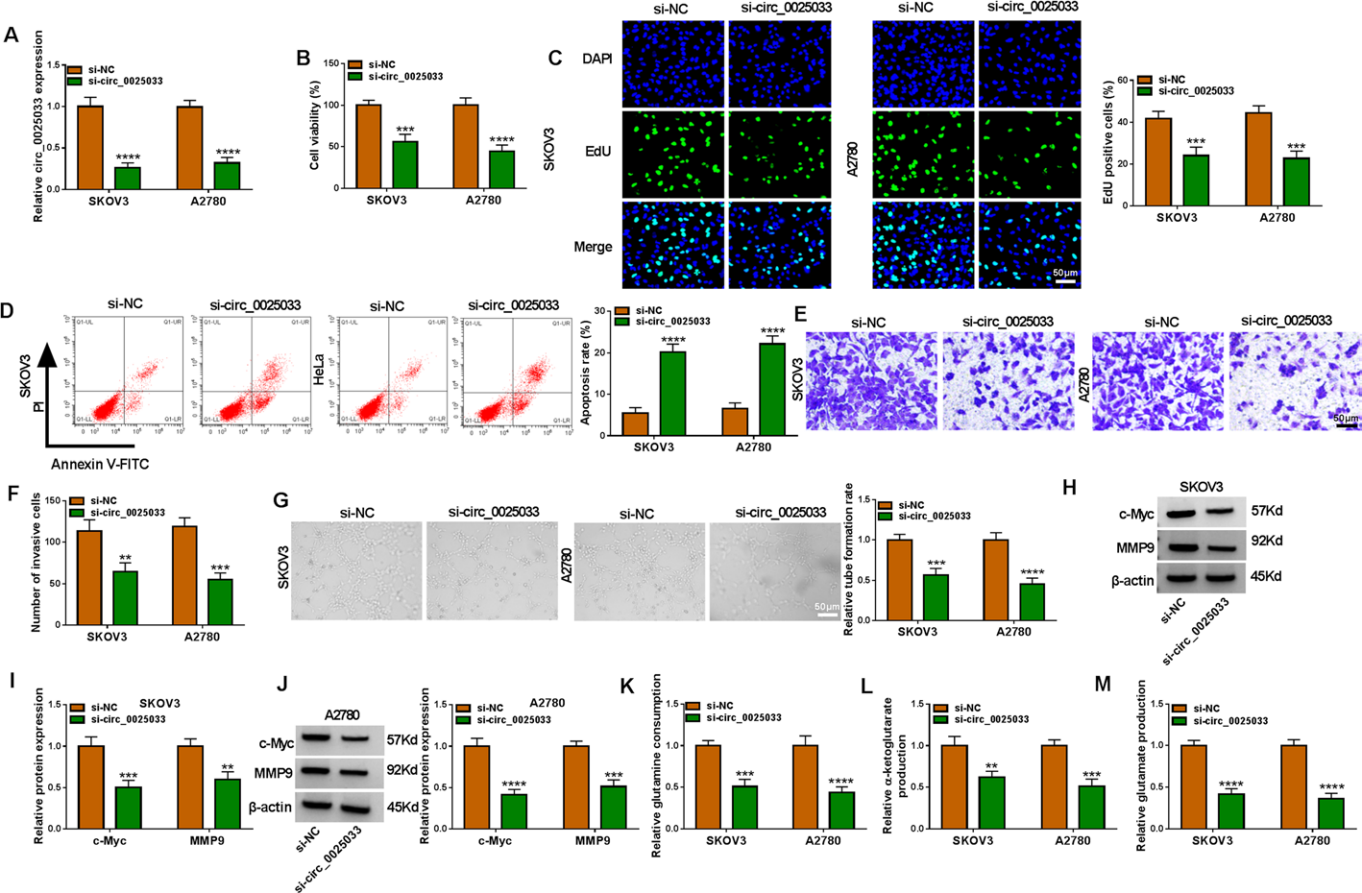
circ_0025033 knockdown hampered the malignant behavior of ovarian cancer cells in glutamine metabolism. **A**–**M** SKOV3 and A2780 cells were transfected with si-NC or si-circ_0025033. **A** qRT-PCR analysis of circ_0025033. **B** and **C** CCK-8 and EdU analysis of cell viability and DNA synthesis. **D** Apoptosis rate analyzed by flow cytometry analysis. **E** and **F** Cell invasion measured by transwell assay. **G** Tube formation assay for evaluating angiogenesis activity. **H**–**J** Western blot analysis of c-Myc and MMP9 levels. **K**–**M** Glutamine consumption, α-ketoglutarate production, and glutamate production measured via glutamine, α-ketoglutarate, and glutamate assay kits, respectively. **P < 0.01, ***P < 0.001, ****P < 0.0001

As expected, circ_0025033 content was diminished in tumor cells via si-circ_0025033 (Fig. 2A). Functionally, circ_0025033 silencing reduced cell viability and DNA synthesis in SKOV3 and A2780 cells (Fig. 2B and C). As shown in Fig. 2D, SKOV3 and A2780 cell apoptosis was increased after circ_0025033 downregulation. Meanwhile, circ_0025033 silencing blocked tumor cell invasion (Fig. 2E and F). Angiogenesis is required for tumor growth and metastasis. Tube formation assay showed that circ_0025033 interference decreased angiogenesis (Fig. 2G). Next, circ_0025033 deficiency reduced levels of proliferation/metastasis-related proteins (c-Myc and MMP9) (Fig. 2H–J). Glutamine, a non-essential amino acid, can be converted into glutamate and then transformed into α-ketoglutarate, which is involved in the tricarboxylic acid cycle to provide energy for cells [27, 28]. Glutamine metabolism is indispensable for tumor development [29]. We found that circ_0025033 silencing reduced glutamine consumption, α-ketoglutarate production, and glutamate production (Fig. 2K–M), suggesting that circ_0025033 downregulation repressed glutamine metabolism. Together, circ_0025033 absence alleviated tumor cell malignancy glutamine metabolism.

### circ_0025033 directly interacted with hsa_miR-370-3p

**Fig. 3:**
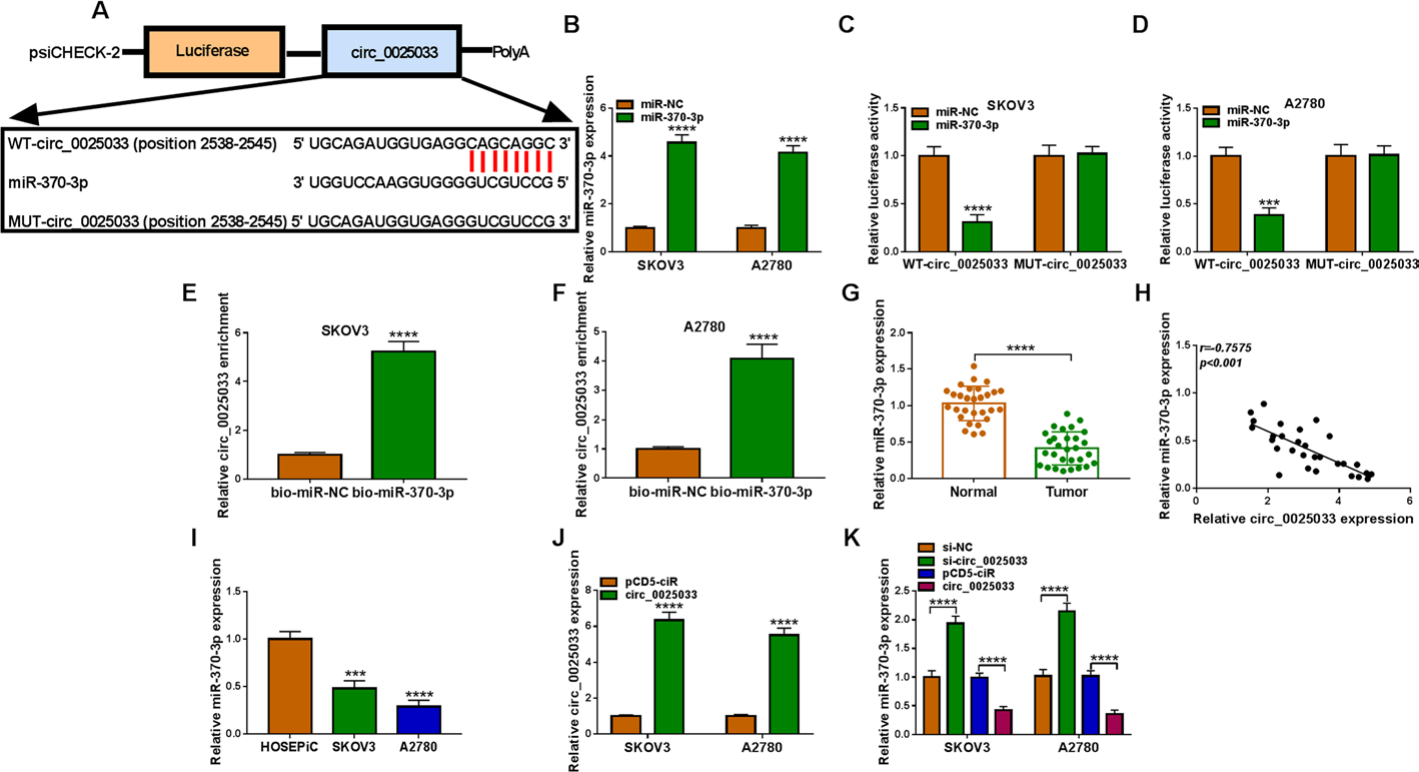
hsa_miR-370-3p was a target of circ_0025033. **A** Circinteractome was used to predict the complementary sequence between hsa_miR-370-3p and circ_0025033. **B** Transfection efficiency of hsa_miR-370-3p in tumor cells. **C** and **D** Their binding was confirmed using dual-luciferase reporter assay in SKOV3 and A2780 cells cotransfected with WT-circ_0025033 or MUT-circ_0025033 and hsa_miR-370-3p or miR-NC. **E** and **F** Their interaction was examined using RNA pull-down. **G** qRT-PCR analysis of hsa_miR-370-3p in tumor tissue. **H** Their correlation was determined via Pearson’s correlation analysis in tumor samples. **I** hsa_miR-370-3p content in HOSEPiC, SKOV3, and A2780 cells. **J** circ_0025033 in cells transfected with pcD5-ciR or circ_0025033 was detected using qRT-PCR. **K** Effect of circ_0025033 deficiency or overexpression on hsa_miR-370-3p content was examined using qRT-PCR. ***P < 0.001, ****P < 0.0001

It has been confirmed that circRNAs could exert their role by interacting with miRNAs [30]. Circinteractome software revealed that circ_0025033 shares binding sites with hsa_miR-370-3p (Fig. 3A), indicating their interaction. Figure 3B shows the overexpression efficiency of hsa_miR-370-3p (Fig. 3B), which exhibited an evident suppression in luciferase activity of WT-circ_0025033, instead of MUT-circ_0025033 (Fig. 3C and D). circ_0025033 was pulled down when using bio-hsa_miR-370-3p rather than bio-miR-NC (Fig. 3E and F). In addition, hsa_miR-370-3p content was downregulated (Fig. 3G), and its level was inversely correlated with circ_0025033 in ovarian cancer tissue (Fig. 3H). Similarly, an obvious decrease of hsa_miR-370-3p in tumor cells was found (Fig. 3I). The significant increase of hsa_miR-370-3p indicated the significant transfection efficiency of pCD-circ_0025033 (Fig. 3J). Next, hsa_miR-370-3p was upregulated via si-circ_0025033, and reduced via circ_0025033 (Fig. 3K), suggesting that circ_0025033 negatively regulates hsa_miR-370-3p expression. Overall, circ_0025033 sequestered hsa_miR-370-3p.

### circ_0025033 knockdown restrained tumor cell malignant phenotypes via regulating hsa_miR-370-3p

**Fig. 4:**
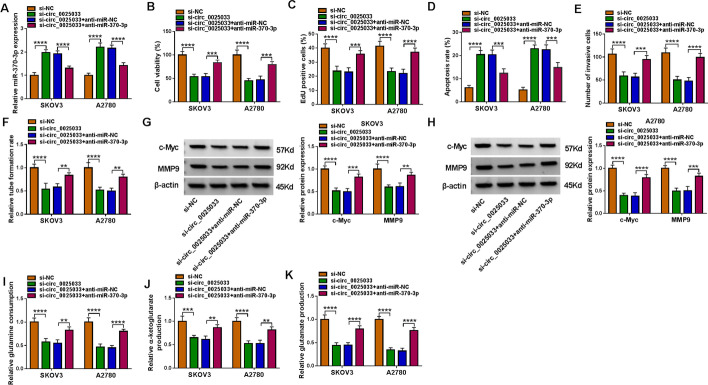
circ_0025033 silencing inhibited ovarian cancer cell malignant behaviors via targeting hsa_miR-370-3p. **A**–**K** SKOV3 and A2780 cells were transfected with si-NC, si-circ_0025033, si-circ_0025033 + anti-miR-NC, or si-circ_0025033 + anti-hsa_miR-370-3p. **A** Expression of hsa_miR-370-3p measured by qRT-PCR. **B** and **C** Cell proliferation detected using CCK-8 assay and EdU assay. **D**–**F** Apoptosis, invasion, and angiogenesis ability assessed using flow cytometry, transwell, and tube formation assays. **G** and **H** Western blot analysis of c-Myc and MMP9. **I**–**K** Glutamine assay kit, α-ketoglutarate assay kit, and glutamate assay kit were utilized to detect glutamine consumption, α-ketoglutarate production, and glutamate production, respectively. **P < 0.01, ***P < 0.001, ****P < 0.0001

We found that circ_0025033 deletion promoted hsa_miR-370-3p expression, while anti-hsa_miR-370-3p abated the effect (Fig. 4A). hsa_miR-370-3p absence mitigated circ_0025033 deficiency-mediated tumor cell viability and DNA synthesis inhibition (Fig. 4B and C). Moreover, circ_0025033 knockdown-induced apoptosis was prevented via hsa_miR-370-3p downregulation (Fig. 4D). In addition, circ_0025033 silencing constrained cell invasion and angiogenesis, and hsa_miR-370-3p inhibition reversed the phenomenon (Fig. 4E and F). Meanwhile, hsa_miR-370-3p reduction might abolish downregulation of c-Myc and MMP9 protein levels caused via circ_0025033 absence (Fig. 4G and H). Further, hsa_miR-370-3p downregulation counteracted the si-circ_0025033-caused reduction in glutamine consumption, α-ketoglutarate production, and glutamate production (Fig. 4I–K). Together, circ_0025033 regulated ovarian cancer cell behaviors by targeting hsa_miR-370-3p.

### *SLC1A5* acted as a direct target of hsa_miR-370-3p

**Fig. 5:**
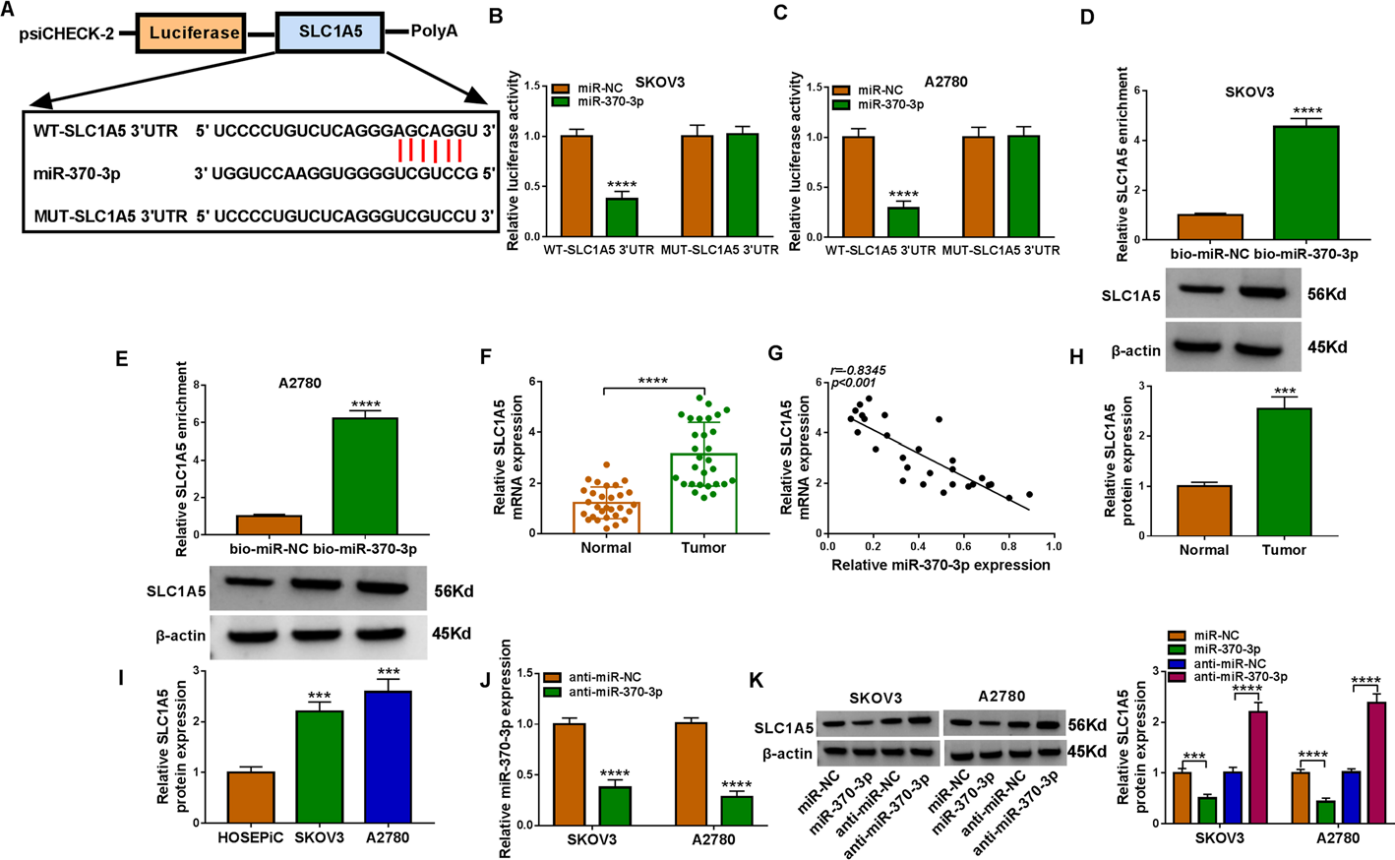
*SLC1A5* was a direct target of hsa_miR-370-3p. **A** Starbase predicted putative binding sites between hsa_miR-370-3p and *SLC1A5*. **B**–**E** Their interaction was confirmed via dual-luciferase reporter and RNA pull-down assays. **F** qRT-PCR analysis of *SLC1A5* mRNA expression in tumor tissues. **G** Their relationship was analyzed via Pearson’s correlation coefficient. **H** and **I** Western blot analysis of *SLC1A5* protein in tissue samples, HOSEPiC cells, SKOV3 cells, and A2780 cells. **J** Transfection efficiency of anti-hsa_miR-370-3p. **K** Effect of hsa_miR-370-3p upregulation or knockdown on *SLC1A5* content was analyzed via western blot. ***P < 0.001, ****P < 0.0001

starBase software revealed that hsa_miR-370-3p harbored some complementary binding sites with *SLC1A5* 3′ UTR (Fig. 5A). hsa_miR-370-3p overexpression strikingly reduced the luciferase activity of WT-*SLC1A5* 3′ UTR (Fig. 5B and C). A higher enrichment of *SLC1A5* was observed in the captured fraction of bio-hsa_miR-370-3p (Fig. 5D and E). Additionally, *SLC1A5* content was significantly reduced in ovarian cancer tissue (Fig. 5F), and its mRNA content was negatively correlated with the hsa_miR-370-3p level (Fig. 5G). Furthermore, *SLC1A5* protein expression was notably enhanced in ovarian cancer tissue and cells (Fig. 5H and I). Transfection of anti-hsa_miR-370-3p reduced hsa_miR-370-3p expression in SKOV3 and A2780 cells (Fig. 5J). In addition, overexpression of hsa_miR-370-3p decreased *SLC1A5* content in tumor cells, and hsa_miR-370-3p absence displayed the opposite effect (Fig. 5K). Taken together, the findings indicate that *SLC1A5* was targeted by hsa_miR-370-3p.

### *SLC1A5* dampened ovarian cancer cell progression

Transfection of si-SLC1A5 reduced *SLC1A5* content in tumor cells (Additional file 1: Fig. S1A). Functionally, deletion of *SLC1A5* notably repressed proliferation, invasion, and angiogenesis while promoting apoptosis (Additional file 1: Fig. S1B–S1F). Moreover, *SLC1A5* knockdown inhibited c-Myc and MMP9 protein expression (Additional file 1: Fig. S1G and S1H). Simultaneously, glutamine consumption, α-ketoglutarate production, and glutamate production were inhibited by downregulation of *SLC1A5* in tumor cells (Additional file 1: Fig. S1I-S1K). These data suggested that *SLC1A5* might be an oncogene in ovarian cancer.

### hsa_miR-370-3p targeted *SLC1A5*

**Fig. 6:**
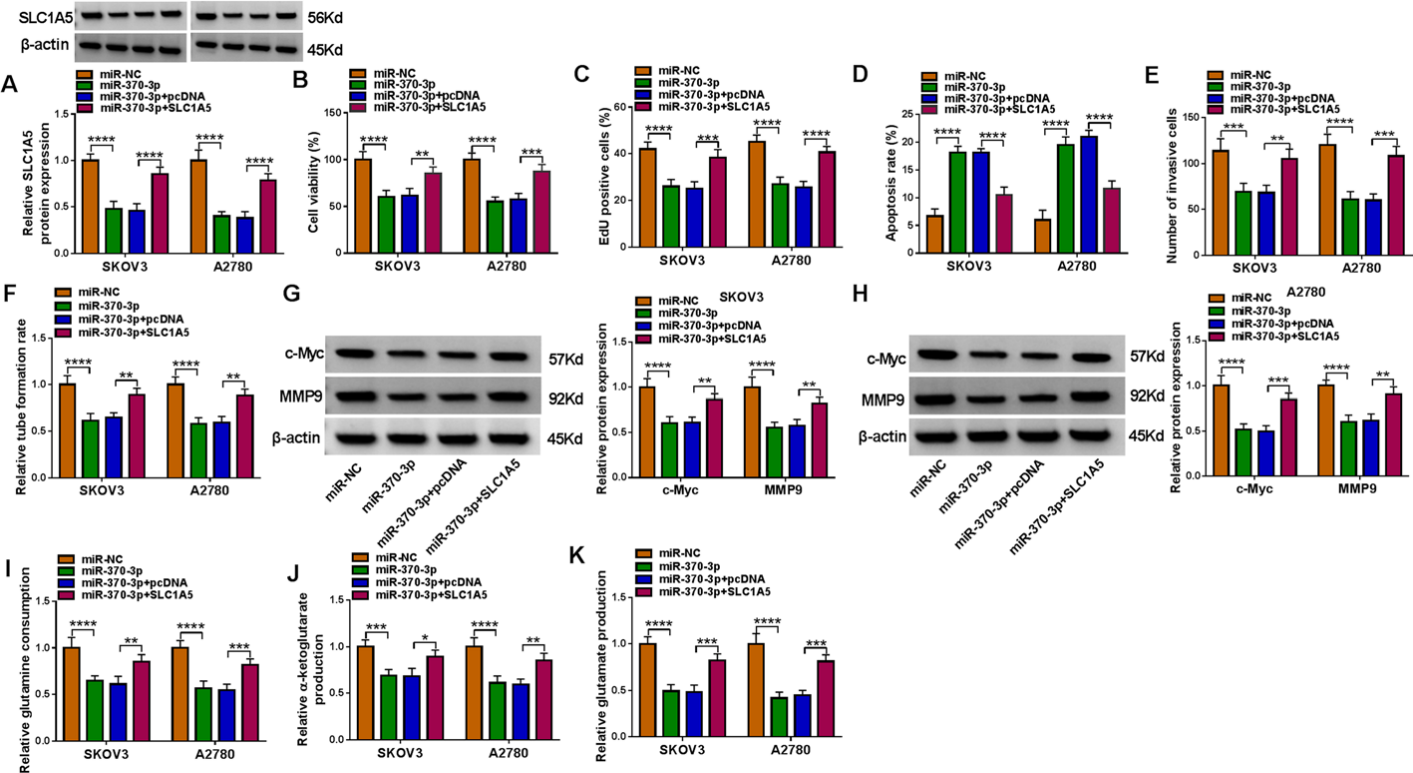
hsa_miR-370-3p negatively regulated *SLC1A5*. **A**–**K** SKOV3 and A2780 cells were transfected with miR-NC, hsa_miR-370-3p, hsa_miR-370-3p + pcDNA, or hsa_miR-370-3p + *SLC1A5*. **A** *SLC1A5* protein expression was examined using western blot. **B** and **C** CCK-8 and EdU assays assessed proliferation ability. **D** Apoptosis, invasion, and angiogenesis capacity was measured using flow cytometry, transwell, and tube formation assays. **G** and **H** c-Myc and MMP9 protein levels were analyzed via western blot. **I**–**K** Glutamine metabolism was evaluated via the corresponding assay kits. *P < 0.05, **P < 0.01, ***P < 0.001, ****P < 0.0001

hsa_miR-370-3p overexpression downregulated *SLC1A5* protein expression, which was rescued by *SLC1A5* upregulation (Fig. 6A). Apart from that, increased hsa_miR-370-3p resulted in a significant suppression in cell proliferation, while increased *SLC1A5* reversed these impacts in tumor cells (Fig. 6B and C). Cell apoptosis was induced, and cell invasion and angiogenesis were inhibited, by hsa_miR-370-3p restoration, which were abated by *SLC1A5* overexpression (Fig. 6D–F). Enhanced hsa_miR-370-3p reduced the protein levels of c-Myc and MMP9, while the re-introduction of *SLC1A5* prevented this reduction (Fig. 6G and H). In addition, glutamine metabolism was decreased by overexpression of hsa_miR-370-3p, which was partly reversed via *SLC1A5* enhancement (Fig.  6I–K). Overall, hsa_miR-370-3p inhibited ovarian cancer cell malignant behaviors via targeting *SLC1A5*.

### circ_0025033 regulated *SLC1A5* expression through sponging hsa_miR-370-3p

As shown in Additional file 2: Fig. S2A and S2B, *SLC1A5* content was dramatically downregulated via circ_0025033 absence, and hsa_miR-370-3p interference recovered the *SLC1A5* content, supporting the regulatory role of the circ_0025033/hsa_miR-370-3p/*SLC1A5* axis.

### Downregulation of circ_0025033 blocked tumor growth in vivo

**Fig. 7:**
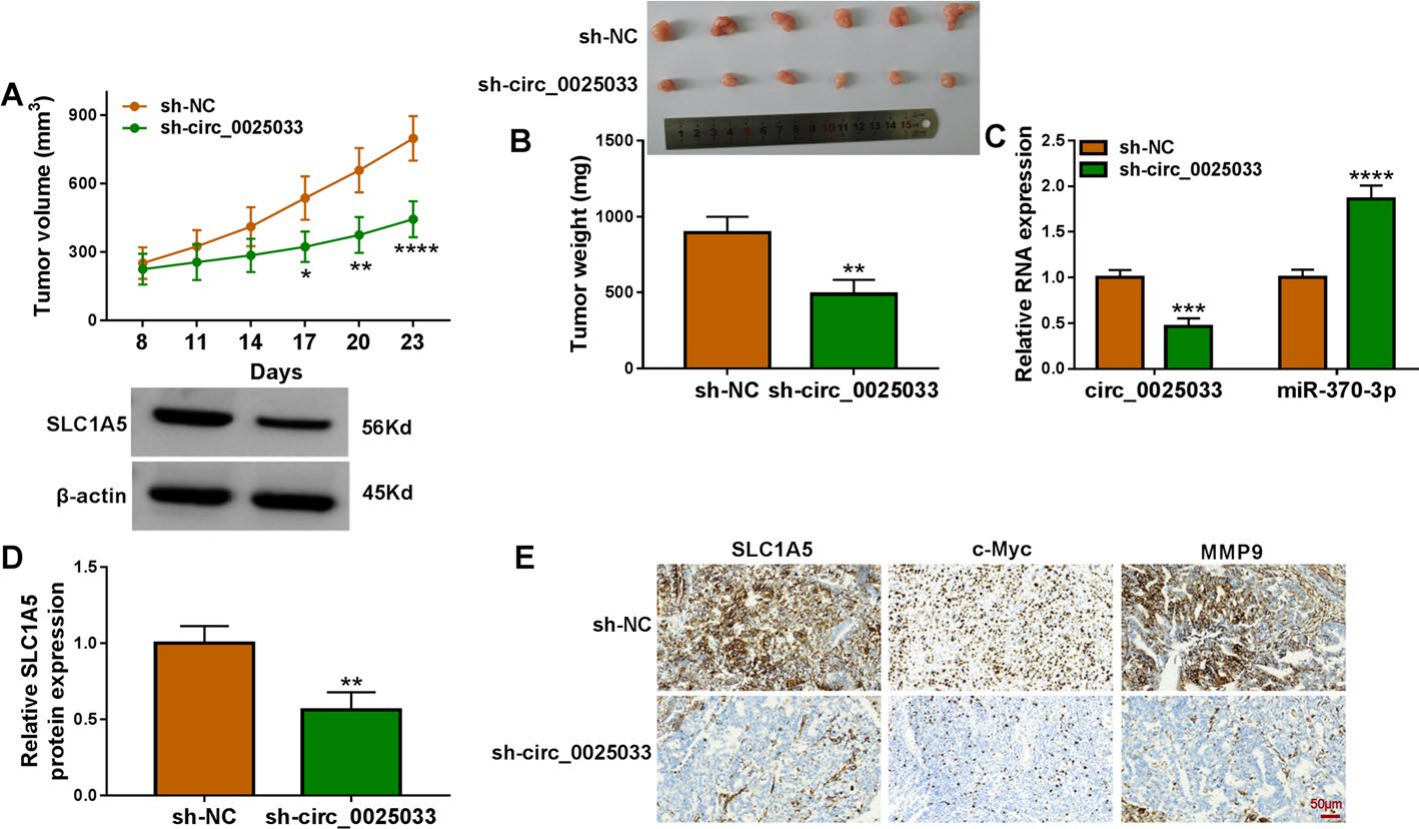
circ_0025033 absence repressed tumor growth in vivo. **A** and **B** Effects of sh-circ_0025033 on tumor volume and weight. **C** circ_0025033, hsa_miR-370-3p, and *SLC1A5* were examined using qRT-PCR and western blot in tumor tissue. **E** IHC analysis was used to detect *SLC1A5*, c-Myc, and MMP9 expression. *P < 0.05, **P < 0.01, ***P < 0.001, ****P < 0.0001

Mouse xenograft models of ovarian cancer were established. As shown in Fig. 7A and B, tumor growth was diminished in the sh-circ_0025033 group (Fig. 7A and B). Apart from that, we confirmed that circ_0025033 expression and *SLC1A5* protein expression were remarkably reduced in the sh-circ_0025033 group, and the hsa_miR-370-3p level was increased (Fig. 7C and D). IHC analysis showed that circ_0025033 silencing suppressed *SLC1A5*, c-Myc, and MMP9 (Fig. 7E). Taken together, circ_0025033 knockdown repressed ovarian cancer growth in vivo.

## Discussion

Patients with ovarian cancer, a gynecologic malignancy, have a short survival time [31]. In this study, circ_0025033 knockdown repressed ovarian cancer cell proliferation, metastasis, angiogenesis, and glutamine metabolism and accelerated apoptosis through the hsa_miR-370-3p/*SLC1A5* axis, which is expected to offer a promising treatment strategy for patients with ovarian cancer.

circRNAs have been shown to be stable in general and aberrantly expressed in various diseases [32]. These characteristics make circRNAs potential therapeutic targets or biomarkers for many diseases, especially cancers. Regarding ovarian cancer, high-throughput sequencing has identified abnormal expression of an increasing number of circRNAs [33, 34]. Nevertheless, the majority of circRNAs in ovarian cancer still need further research. circ_0025033 has been shown to promote cell invasion by targeting the miR-1304/miR-1231 axis in papillary thyroid cancer [35]. Moreover, circ_0025033 was upregulated, and its knockdown inhibited ovarian cancer cell viability and metastasis through targeting the miR-330-5p/KLK4 axis [36]. In addition, Hou and Zhang report that circ_0025033 downregulation suppressed colony formation ability, mobility, and glycolysis metabolism in ovarian cancer cells via regulation of the LSM4/miR-184 axis [18]. However, the roles of circ_0025033 in angiogenesis and glutamine metabolism have not been reported. In line with previous research, high circ_0025033 levels in tumor specimens and cells were observed. Moreover, circ_0025033 deficiency limited tumor malignant phenotypes, indicating its promoting effect in ovarian cancer.

Accumulating reports have indicated that circRNAs in the cytoplasm function as miRNA sponges, resulting in changes of target gene expression [23]. In this research, circ_0025033 was predominantly located in the cytoplasm. Hence, circ_0025033 was a hsa_miR-370-3p sponge. Cumulative evidence indicates that hsa_miR-370-3p has a strong ability to modulate tumor development. When hsa_miR-370-3p level is reduced, its increase might inhibit the development of bladder cancer [37], papillary thyroid carcinoma [38], gliomas [39], and acute myeloid leukemia [40]. However, hsa_miR-370-3p expression is enhanced and acts as a tumor-promoting miRNA in gastric carcinoma [41] and breast cancer [42]. In terms of ovarian cancer, hsa_miR-370-3p suppression abated circAGFG1 interference-mediated ovarian cancer cell growth and migration [43]. In addition, hsa_circ_0061140 absence repressed ovarian cancer cell metastasis through sponging miR-370 [44]. Herein, hsa_miR-370-3p showed a low level in ovarian cancer tissue samples and ovarian cancer cells. Rescue assays revealed that suppression of hsa_miR-370-3p counteracted circ_0025033 deficiency-triggered ovarian cancer cell proliferation, apoptosis, metastasis, angiogenesis, and glutamine metabolism inhibition, indicating that circ_0025033 promoted ovarian cancer cell progression via downregulating hsa_miR-370-3p.

Online software Starbase indicated that *SLC1A5* may be an hsa_miR-370-3p target. *SLC1A5*, a glutamine transporter, can control glutamine uptake and is essential for tumor growth [45, 46]. *SLC1A5* plays as a vital role in prostate cancer [47], gastric cancer [48], lung cancer [49], and esophageal cancer [50]. Importantly, Huang and her colleagues stated that upregulation of miR-122-5p inhibited ovarian cancer process via targeting *SLC1A5* [26]. High *SLC1A5* levels were associated with poor prognosis for patients with ovarian cancer [51]. In this research, *SLC1A5* silencing inhibited ovarian cancer cell malignant behaviors, indicating a cancer-promoting role of *SLC1A5* in ovarian cancer cells. Furthermore, *SLC1A5* upregulation could abrogate hsa_miR-370-3p-triggered anti-ovarian cancer. Mechanistically, circ_0025033 could regulate *SLC1A5* expression in ovarian cancer cells via binding to hsa_miR-370-3p. Consistently, tumor growth in this research was also suppressed via circ_0025033 knockdown in vivo.

In conclusion, circ_0025033 interference repressed ovarian cancer cell malignant behaviors and glutamine metabolism via the hsa_miR-370-3p/SLC1A5 axis, indicating an underlying therapeutic target for the tumor.

## Supplementary Information

**Additional file 1: Fig. S1.** *SLC1A5* and circ_0025033 had similar roles in ovarian cancer. (A-K) SKOV3 and A2780 cells were transfected with si-NC or si-*SLC1A5*. (A) Western blot analysis of *SLC1A5* content. (B-E) Proliferation, apoptosis, and invasion were assessed using CCK-8, EdU, and flow cytometry assays, respectively. (F) Angiogenesis ability was evaluated using tube formation assay. (G and H) Western blot analysis of c-Myc and MMP9. (I-K) Glutamine metabolism was analyzed using special kits. ***P < 0.001, ****P < 0.0001.

**Additional file 2: Fig. S2.** Circ_0025033 sponged hsa_miR-370-3p to regulate *SLC1A5* expression. (A and B) Effects of si-circ_0025033 and anti-hsa_miR-370-3p on *SLC1A5* content were monitored using western blot. **P < 0.01, ***P < 0.001, ****P < 0.0001.

## Data Availability

Not applicable.

## Abbreviations

circRNAs: Circular RNAs
ncRNAs: Noncoding RNAs
*FOXM1*: Forkhead box M1
miRNA: MicroRNA
ceRNAs: Competitive endogenous RNAs
*SLC1A5*/ *ASCT2*: Solute carrier family 1 member 5
