# Supplementary figures and images for: circ_0025033 promotes ovarian cancer development via regulating the hsa_miR-370-3p/SLC1A5 axis
Source: Cell Mol Biol Lett. 2022 Oct 22;27:94. doi: 10.1186/s11658-022-00364-2 (PMC9588225; doi:10.1186/s11658-022-00364-2)

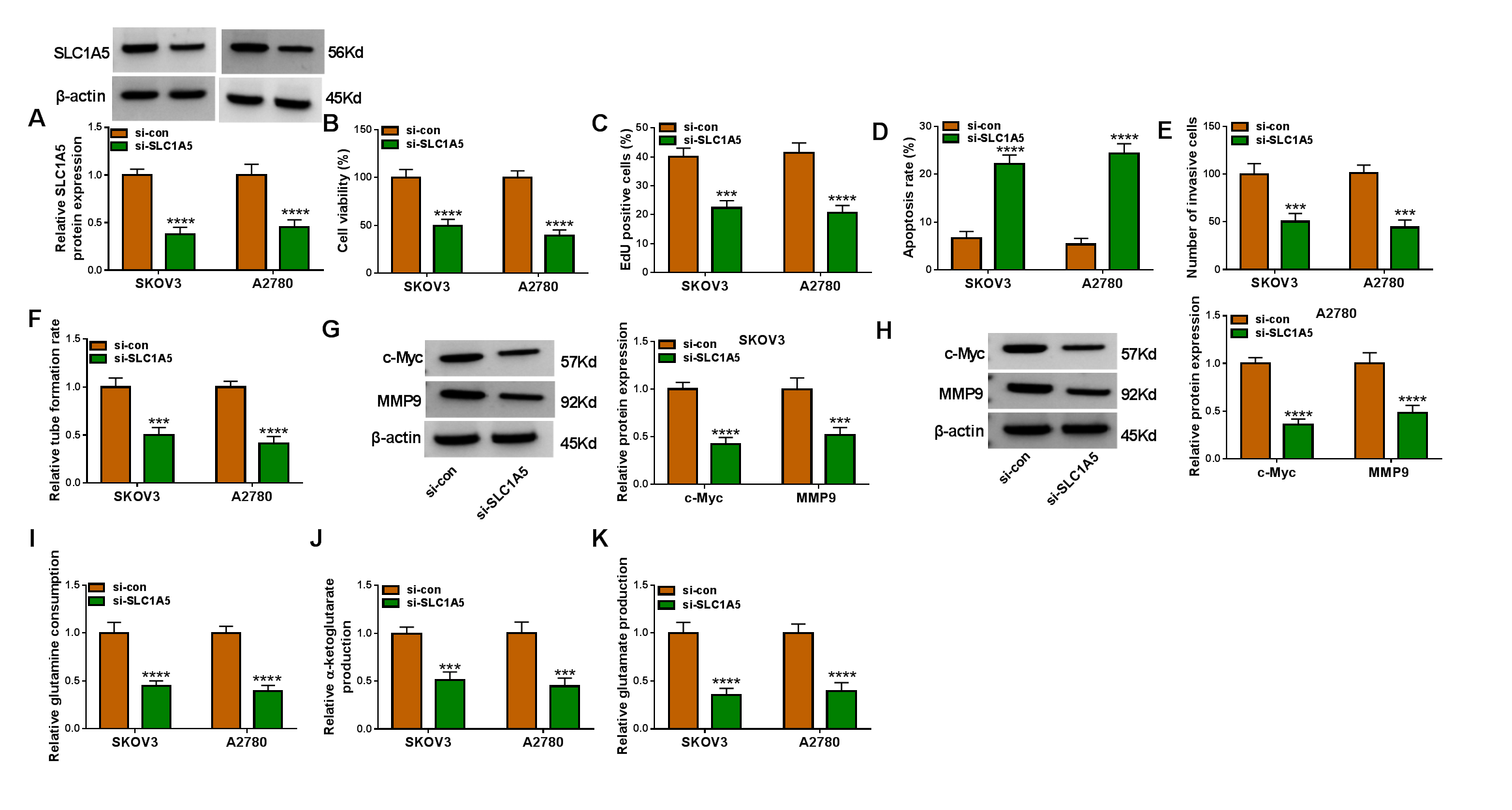

Supplement: Supplementary file 1 — Additional file 1: Fig. S1. SLC1A5 and circ_0025033 had similar roles in ovarian cancer. (A-K) SKOV3 and A2780 cells were transfected with si-NC or si-SLC1A5. (A) Western blot analysis of SLC1A5 content. (B-E) Proliferation, apoptosis, and invasion were assessed using CCK-8, EdU, and flow cytometry assays, respectively. (F) Angiogenesis ability was evaluated using tube formation assay. (G and H) Western blot analysis of c-Myc and MMP9. (I-K) Glutamine metabolism was analyzed using special kits. ***P < 0.001, ****P < 0.0001. [file 11658_2022_364_MOESM1_ESM.tif]

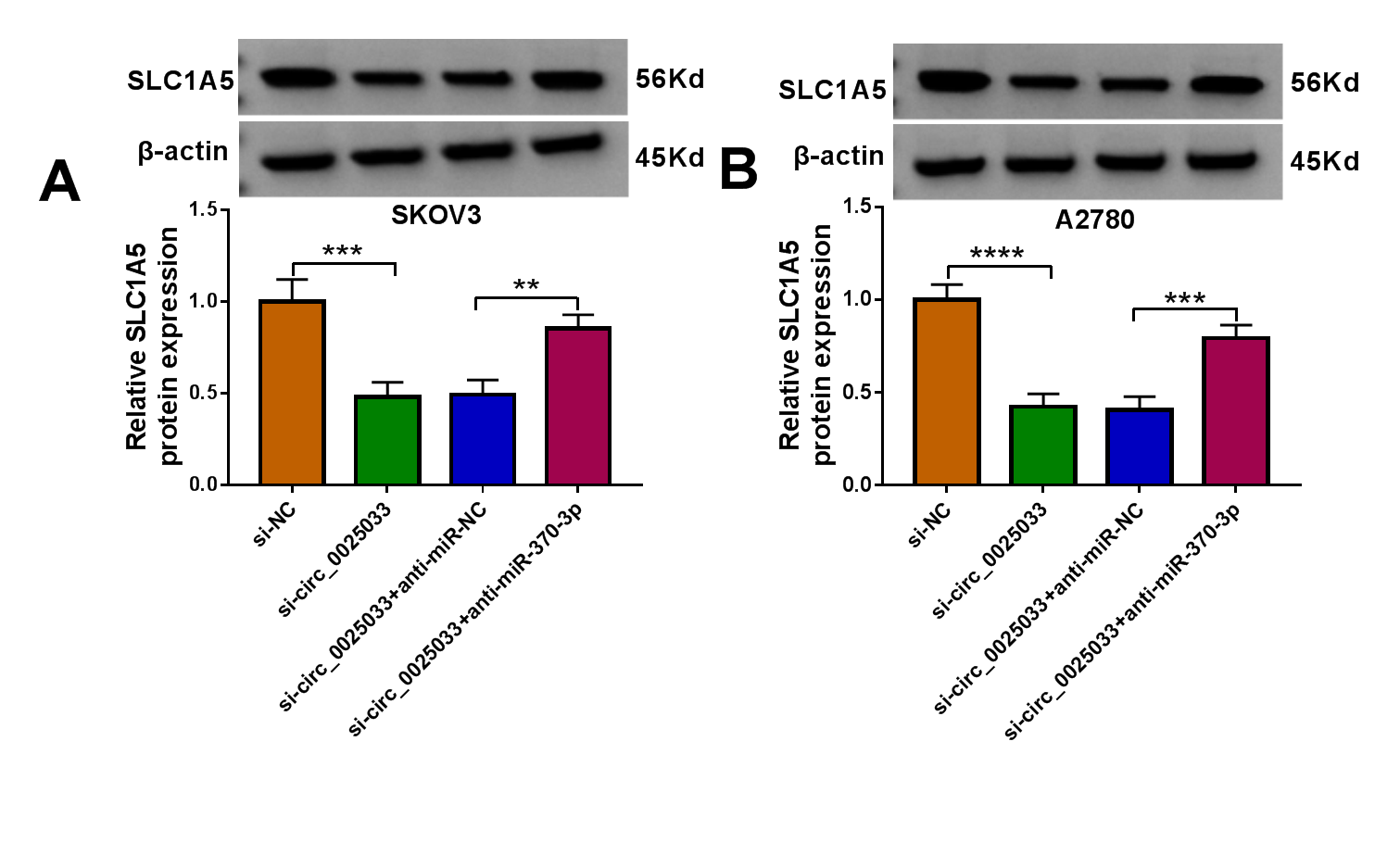

Supplement: Supplementary file 2 — Additional file 2: Fig. S2. Circ_0025033 sponged hsa_miR-370-3p to regulate SLC1A5 expression. (A and B) Effects of si-circ_0025033 and anti-hsa_miR-370-3p on SLC1A5 content were monitored using western blot. **P < 0.01, ***P < 0.001, ****P < 0.0001. [file 11658_2022_364_MOESM2_ESM.tif]
